# Stories that matter: a qualitative study of general practitioners’ reflections and experiences of exploring patients’ impactful life stories

**DOI:** 10.1080/17482631.2025.2454043

**Published:** 2025-01-23

**Authors:** Marianne Rønneberg, Bente Prytz Mjølstad, Lotte Hvas, Linn Okkenhaug Getz

**Affiliations:** a Tingvoll Healthcare Centre, Norway and General Practice Research Unit, Department of Public Health and Nursing, Norwegian University of Science and Technology (NTNU), Trondheim, Norway; b General Practice Research Unit, Department of Public Health and Nursing, Norwegian University of Science and Technology (NTNU), Trondheim, Norway; c The Research Unit for General Practice and Section of General Practice, Department of Public Health, University of Copenhagen, Copenhagen, Denmark

**Keywords:** General practice, trauma-informed care, narratives, epistemic injustice, pattern recognition, inductive foraging

## Abstract

**Purpose:**

There is solid evidence of the impact of life experiences on health. Yet, knowledge of how general practitioners (GPs) relate to patients’ stories of such experiences is sparse. This study explored GPs’ reflections and experiences concerning managing potentially impactful patient stories.

**Methods:**

We conducted four focus group interviews among Norwegian and Danish GPs and analysed them using Reflexive thematic analysis.

**Results:**

Three main themes were developed. First, GPs apply various strategies to recognize and unfold impactful stories. Second, they attribute diverse purposes to engaging with these stories, from viewing them as instrumentally useful to recognizing their intrinsic value. These views influence GPs’ objectives and strategies when managing impactful stories. The instrumental utility approach can lead to an unfair dismissal of impactful stories. Finally, the commitment of some of the GPs to patients’ impactful stories is considered fulfilling and highly satisfactory but also associated with external resistance.

**Conclusions:**

Patients and GPs encounter difficulties in addressing impactful stories, which resonate with the theory of epistemic injustice. Nevertheless, engaging with these stories is vital for providing ethically grounded and meaningful primary care. The paper proposes strategies and a conceptual framework to support work with impactful stories in clinical practice.

## 1. Introduction & background

It is well-documented that life experiences and health are reciprocally interconnected (Felitti et al., 1998; Getz et al., 2011). Adverse life experiences increase the risk of health problems from a life course perspective (Felitti, 1998; Halfon et al., 2014). In the general practice literature, acknowledgment of how patients’ stories of life experiences matter in a literal (i.e., biological) sense can be traced back to several influential academic thinkers, including the philosopher of general practice/family medicine Ian McWhinney (McWhinney, 1996, 2000), social epidemiologist Nancy Krieger (Krieger, 2005) and primary healthcare services researcher Barbara Starfield (Starfield, 2011). Hence, insight into life experiences that impact the health of patients is relevant to medical doctors, and in particular to GPs who care for their patients over time (Cassell, 2010; McWhinney, 1996, 2000; Mjølstad, 2015; Peabody, 1927).

During the last 10–15 years, an approach called *Person centred medicine* (PCM) has gained momentum internationally. PCM is influenced by the bio-psycho-social model and the patient-centred method (Louw 2017), as a further extension of the patient-centred method (Levenstein et al., 1986) in response to a call for a more holistic, humane, and context-sensitive practice (Dowrick, 2017; Kirkengen et al., 2012; Miles & Mezzich, 2011; Starfield, 2011). There is no consensus on the definition of PCM (Louw, 2017). According to our apprehension, it acknowledges that the medical understanding of causality and treatment options—its clinical evidence base—should include not only biomedical knowledge but also insights derived from qualitative methods and patients’ narratives. PCM represents a holistic approach that is scientifically grounded and can address complex causality, representing a unique ontology (Anjum, 2016; Anjum et al., 2020).

Along similar lines, *generalism* has received increased attention as an essential clinical approach or philosophy in general practice/family medicine. Defined as “expertise in whole person care” (Reeve et al., 2013), generalism “describes an approach to care which is person-, not disease-focused, continuous, not episodic; integrates biotechnical and biographical understanding of illness” (Reeve et al., 2013). Wise integration (Muench, 2018) of both biomedical and experiential dimensions of an individual’s situation lies at the core of generalist competency (Lynch et al., 2023; Reeve, 2023).

In general practice consultations, patients share accounts of their lived experiences as *stories* or *narratives*. The two terms are often used interchangeably (Launer, 2018) as we to some extent will do in this paper. Nevertheless, it is important to highlight a nuanced differentiation. Compared to “story”, the term “narrative” more explicitly invites interpretation, questioning, and co-creative adjustments in perspective (Greenhalgh & Hurwitz, 1999; Launer, 2002). The present study focuses on stories/narratives that significantly impact persons’ health and thereby carry medical relevance. In this paper, we will refer to such stories as “impactful” (see Box 1).

**Box 1.** Medically impactful stories

We use the term *impactful stories* as a shorthand for *stories that impact the patient’s/person’s health in a clinically significant manner, down to a deep biological level*. This refers to life events and experiences with causal relevance for the individual’s susceptibility to health problems and suffering in a wide sense, encompassing both physical and mental dimensions of health. Here, we lean on Eric Cassell’s definition of suffering as “the state of severe distress associated with events that threaten the intactness of the person”. Suffering is a subjective experience. It “can occur to any aspect of the person, whether in the realm of self, body, or family or the relation with a transpersonal, transcendent source of meaning”. Another important aspect of suffering is that it persists until “the threat of disintegration has passed or until the person can be restored in some other manner” (Cassell, 1998). When we use the term *impactful story*, we would like to clarify that neither the doctor nor the patient can know in advance if a story will impact health. Presumptions regarding a story’s medical relevancecan be based on empirical research from several disciplines, demonstrating different pathways through which life experiences affect the development of health and disease (Getz et al., 2011). Australian GP researcher Johanna Lynch has described relevant examples of impactful stories as “Adverse childhood experiences with proven physiological impact” (Lynch, 2020, p. 61) with reference to key publications in the field (Felitti et al., 1998; Teicher & Parigger, 2015). Lynch outlines three applicable types of such experiences: *Disconnection* in the sense of being rejected and excluded. This includes both physical and emotional neglect and abuse, verbal as well as non-verbal. The second category involves physical and sexual *violence and abuse*, being a witness to violence between parents or siblings, and peer physical bullying. The last category is called the *missing parent* and points to parents who are absent, intoxicated, hospitalized, or incarcerated (Lynch, 2020, p. 61).

Discussions of the when, the why and the how to address and work with patient stories/narratives in clinical settings are ongoing. However, since the days of Ian McWhinney’s writings, formal academic interest in the topic has been surprisingly limited. Nonetheless, essential publications do exist. One such publication is the book Narrative-based Primary Care published by GP writer John Launer (Launer, 2002). He states that “One can (…) see medicine and primary care especially, as essentially a story-making activity”(Launer, 2006, p. 342). Launer describes three different levels of what he calls narrative work in general practice. The most basic involves the listening associated with general medical history taking. On the second level, the GP listens attentively to the patient’s spontaneous and often somewhat fragmented narrative to help them “give coherence to their own story.” The third level represents a more explicit therapeutic stance as it “involves questioning the patient in a way that explores new meaning which may make a difference to the patient” (Launer, 1998). At this level, a doctor and a patient are in the position to co-create a shared understanding of a problem or situation, which, ideally and over time, will result in “stories that work better” for the patient in the sense that they contribute to maintenance or restoration of health (Launer, 2002). Here, Launer aligns with the works of other influential thinkers, including physician Howard Brody and sociologist Arthur Frank who emphasize the mutual clinical relationship as a prerequisite for patients to formulate, share, and restructure their illness stories as part of a healing process (Brody, 1994; Frank, 1998).

**Figure 1.**
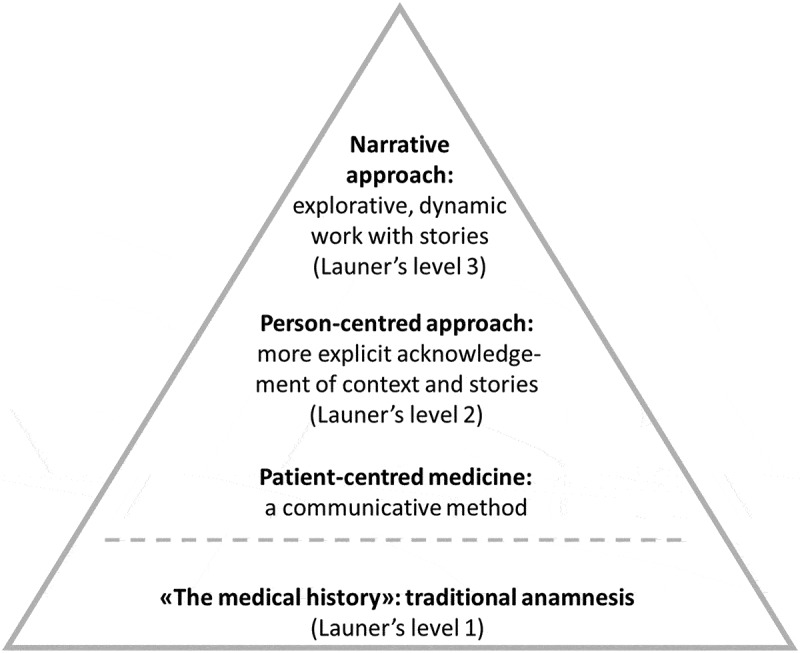
Tentative illustration of how patients’/person’s stories can be acknowledged and incorporated in clinical work at different levels.

It is becoming increasingly clear that knowing patients’ impactful stories may contribute to more effective and temperate healthcare. Insight into patients’ life worlds helps GPs understand and ease patients’ suffering (Epstein & Back, 2015). If impactful stories are interpreted as comprehensible and manageable, they strengthen a person’s *sense of coherence* and build resilience. A recent study demonstrating how being able to tell an integrated story of stressful experiences reduces levels of biological parameters leading to cell aging (telomere shortening) supports just that (Mason et al., 2019). Additionally, GPs should consider adverse childhood experiences, stories of growing up in poverty or socially deprived areas, and exposure to natural and man-made disasters (Burns et al., 2017) when assessing patients’ risk for developing allostatic overload (McEwen & Getz, 2013; Tomasdottir et al., 2015) and disease (Felliti et al., 1998). Attentiveness to these factors in patients' biographies is also crucial when trying to make sense of phenomena like multimorbidity and medically unexplained symptoms (Barnett et al., 2012; Kirkengen et al., 2016; Tomasdottir et al., 2015). Exploring and recognizing the links between unexplainable symptoms and stories of childhood violation reduces unnecessary referrals to numerous somatic examinations and eventually leads to less overdiagnosis (Kirkengen, 2001; Getz, 1999). In Figure 1, we have attempted to depict how patients’ impactful stories can be integrated into clinical work with increasing levels of sophistication (Figure 1).

According to the outline above, evidence of the links between life experiences and health and the advantages of knowing patients’ impactful stories are well documented. Yet, only a few empirical studies provide insights into the relevance of such stories in general practice. In 1997, a survey of Norwegian GPs revealed that less than half of the consultations identified relevant psychosocial issues like lived realities and life stories, highlighting that many such problems remained unaddressed (Gulbrandsen et al., 1997). In more recent studies performed by our group, GPs in Norway were surprisingly often unaware of important adverse life experiences in their patients’ biographies, despite long doctor-patient relationships (Mjølstad, 2015; Mjølstad et al., 2013). Furthermore, it appears that GPs sometimes express uncertainty and ambivalence regarding the medical relevance of such stories (Rønneberg et al., 2022). Some of the GPs included in the latter study took a *confident-accepting stance to stories*, characterized by explicit acknowledgment of the medical relevance of patients’ stories of painful and adverse experiences. In contrast, the remaining participating GPs represented an insecure and *ambivalent stance*, typically associated with a *conditional* acceptance with defined premises for when they found it within their professional mandate to open for patient stories of adverse and painful life experiences.

As these empirical studies indicate, GPs do not consistently engage with patients’ impactful stories. Further, little is known about how and when GPs do explore and attend to such stories. This study aims to contribute to the field by investigating GPs’ experiences and reflections concerning managing potentially impactful patient stories. We include perspectives from typical GPs and GPs with a particular interest in person-centred medicine.

## 2. Methodology

To achieve our research objectives, we carried out a descriptive qualitative study. We recognize that such studies involve researchers’ interpretations (Braun & Clarke, 2021a). Our study has an experiential focus, in the sense that language reflects reality and is regarded as a tool for communicating thoughts, feelings, and experiences (Braun & Clarke, 2022).

To elicit a wide range of perspectives, we conducted focus group interviews among what we considered “typical” GPs, and GPs who share a special interest in person-centred medicine.

We chose focus group interviews as they are considered particularly well-suited to studying attitudes and experiences (Kitzinger, 1995), and to explore fields where little is known in advance (Morgan, 1998). Besides, we wanted to accommodate the dynamics that focus groups could elicit, with space for a wide range of reflections, including tentative and conflicting views (Kitzinger, 1995). Our approach was inspired by the semi-structured lifeworld interview, a phenomenological approach that helps in understanding “themes of the lived everyday world from the subjects’ perspectives” (Brinkmann & Kvale, 2015, p. 27).

### 2.1. Sampling and recruitment

In the first stage of this study, we purposively recruited what we considered to be “typical and dedicated” regular GPs in Norway for three focus group interviews (focus groups 1–3). Beyond being regular GPs, these participants supervised final-year medical students attending in-service training at university-affiliated healthcare centres. Such supervision only takes place six weeks every year, so except for the GP’s administrative university affiliation, we assumed these GPs would not differ from other dedicated Norwegian regular GPs. They were invited to participate in the focus groups while participating in voluntary courses for GP tutors organized by the university in 2016/17. These GPs were unknown to the researchers, except for some brief contacts in other professional settings. Data from focus groups 1–3 has previously formed the basis for a published paper about GPs’ perceptions of the medical relevance of stories of painful and adverse life experiences, that we refer to in the introduction (Rønneberg et al., 2022). In the present study, we applied the same empirical material, but this time we wanted to explore another aspect of the data; GPs’ experiences and reflections on managing patients’ impactful stories.

In the second stage of the study, we invited an established group of Danish GPs who are members of a general practice Think tank to participate (focus group 4). The group was established in 2014, comprising 15 GP specialists. In previous internal meetings, the think tank had focused on exploring “the discipline of general practice” and delved into discussions of how GPs can improve their practice by developing person-centred medicine. Knowing this, we assumed that a focus group interview with this group might open perspectives that would expand and/or differ from the three preceding focus group discussions. Therefore, a supplementary fourth focus group interview was conducted during one of the Danish group’s regular gatherings in 2018. To our knowledge, no comparable group could have been identified in Norway. Choosing a GP group from Denmark was also appropriate since the health care systems in the two countries are organized similarly, with contract GPs providing a wide range of generalist services while also serving a key role as gatekeepers to specialist health care. The participating GPs from both countries had working contracts with local municipalities. For the purpose of this paper, we choose to call the participants in the three first focus groups *regular GPs* and GPs from the fourth focus group *think tank GPs*, respectively.

### 2.2. Data collection & analysis

The first three focus group interviews were conducted with Norwegian *regular GPs* as described in the previously mentioned paper (Rønneberg et al., 2022). MR and BPM moderated the focus group discussions which lasted 70–90 minutes. Each group consisted of GPs working in urban and rural areas. A total of 11 men and 7 women aged 32–69 participated. Fourteen of the GPs were certified as specialists in general practice.

The *Think tank GP group* encompassed 10 of its 15 members, all specialists in general practice aged 48 to 66 years, 5 men and 5 women, serving urban and rural areas.

Before the Think tank group discussion started, MR set the scene by summarizing our main impressions from the three Norwegian focus groups, in particular the two main stances on the medical relevance of patients’ stories of painful and adverse life experiences. LG and BPM moderated the subsequent focus group discussion. The moderators used some of the questions from the same semi-structured interview guide we applied to the three focus groups among Norwegian GPs (see supplementary material). Key topics included how GPs get access to patients’ stories, and characteristics of situations in which GPs sense that the patient might have a story that could be relevant. The discussion lasted 120 minutes.

All four focus group interviews were audio recorded and transcribed verbatim into Norwegian by MR, who was also responsible for taking field/reflection notes during and after the sessions.

We applied *reflexive thematic analysis* to our data (Braun & Clarke, 2019, 2022; Terry et al., 2017). Reflexive thematic analysis seeks to reveal patterns of meaning across the data (Braun & Clarke, 2022). Typically, codes and themes are developed inductively (Terry et al., 2017). Reflexive thematic analysis is among others well-suited to analysing data derived from research questions related to people’s experiences and perceptions. Additionally, it can be applied to heterogeneous samples like ours (Braun & Clarke, 2021a).

All four authors participated in the analysis, following the six analytic steps described by Braun and Clarke (Braun & Clarke, 2006). First, we familiarized ourselves individually with the data while making observational notes. In the next step, we compared and discussed our observations and notes. Codes were then generated inductively informed by the research questions. The codes were mostly descriptive (semantic). We formulated potentially overarching themes based on these codes. In this process, we also conducted what Guest et al refer to as a “purely qualitative (interpretive thematic) comparison” (Guest et al., 2012). The idea behind that approach is for investigators to seek themes only present in some groups under study or themes expressed differently between groups (Guest et al., 2012). During this process, we noticed that some themes only appeared in focus group 4. We refer to the differences in the results section. Finally, we completed the analysis by returning to the transcripts to check that the identified themes represented the most relevant subjects discussed during the interviews. Quotes included were translated into English by MR and validated by the other authors.

### 2.3. Quality criteria

To ensure rigour and transparency in our research, we consulted Braun and Clark’s twenty guiding questions to assess our thematic analysis research quality (Braun & Clarke, 2021b)

As moderators of the focus groups (MR and BPM focus groups 1–3, BPM and LG focus group 4), we tried to let the participants speak as freely as possible. Questions were open-ended, and we knew the importance of setting aside our preconceptions. We took field notes immediately after each interview. The notes included participants’ non-verbal responses. We described responses we found especially interesting or surprising and paid particular attention to such aspects of our data when analysing the material.

We applied principles of *information power* (Malterud et al., 2021) to guide the sample size of our study. The relatively narrow aims of the study, combined with the purposive sampling strategy, robust dialogues during the focus group interviews, and the diversity of participants’ perspectives, suggest that the data’s *information power* is sufficient.

As a brief reflexive statement, the first author (MR) has worked as a GP in a rural area for nearly 20 years and is also a GP tutor. BPM is an experienced GP and senior qualitative researcher. LG practiced earlier as a GP and is now a Professor of Behavioural Sciences. LH has worked as a GP in Denmark for decades and is also an experienced qualitative researcher. As professionals, the authors recognize that patients’ past and current medical problems and their stories of painful and adverse experiences are related. LG, BPM, and LH have also conducted research into that subject.

## 3. Ethics approval and consent to participate

Written consent was obtained from all focus group participants to participate in the interviews/analyses. Transcripts were anonymized and all data was safely secured according to Norwegian and Danish regulations. Before conducting the focus group interviews, we consulted the Regional Committee for Medical and Health Research Ethics (REK Midt). No formal application was required (subsequently verified with reference 2018/1193). The Norwegian Centre for Research Data (NSD) was also consulted. Focus group interviews among professionals that did not elicit sensitive personal information or health data did not need further ethical approval. The Danish GPs were informed that although their accounts would be anonymized, the think tank group as such might be recognizable. In Denmark, focus group interviews among professionals do not require ethical approval (Danish Research Ethics Committees, 2024).

## 4. Results

From the analysis of the empirical material, we developed three main themes describing the participating GPs’ experiences and reflections on how they attend to patients’ stories that impact health. First, the GPs use various strategies to recognize and unfold such stories: sensory awareness, recognition of patterns and pattern deviations, and a broad but swift exploration of the patient’s situation and life circumstances. Second, they attribute diverse purposes to engaging with these stories, from viewing them as instrumentally useful to recognizing their intrinsic value. These views influence GPs’ objectives and clinical management strategies. Finally, the think tank GPs described a particular *mindset;* that is: how they commit to impactful stories as persons. The study also highlights how the commitment of some of the GPs to patients’ impactful stories is considered fulfilling and highly satisfactory but also associated with external resistance.

### 4.1. Accessing impactful stories: a wide range of approaches

The first main theme describes the GPs’ skills and strategies to recognize and unfold impactful stories. It includes deliberate sensory awareness, the ability to recognize typical patterns, the discernment of deviations from these patterns, and conducting a broad exploration. The think tank GPs specifically outlined the latter strategy.

#### 4.1.1. Attentive presence

The GPs highlighted how their attentive presence *sensing patients’ emotions or sensing and picking up communicative cues and hints* during consultations can serve as leading threads to impactful stories. These cues encompass nonverbal communication such as hesitation, lowered voice, slow speech, and tearfulness.

Some participants noted that patients might test the doctor’s response by vague or impersonal statements (hints), such as mentioning a relative’s difficulties in life. If the GP responded openly, the patient would often share a personal, painful story. An experienced GP in focus group 2 described this as “sending up trial balloons”. The *GPs’ own perceptions and feelings could also guide* them to suspect that the patient had an impactful story. Some of the regular GPs mentioned relying on their “gut feeling”. One noted a subtle sense that the patient was burdened. An example of how a patient’s presence can be reflected in a GP’s inner perception in a notable and informative manner is illustrated in the following dialogue between one of the regular GPs (focus group 2) and the interviewer (I):

GP: I can often sense if patients feel unsafe.

I: What do you mean by that?

GP: It’s something about their appearance. It’s the body language, the way the patients sit, if they dare look you properly in the eyes, and if they maintain eye contact. It’s the way they express themselves.

I: Almost like bodily sensations?

GP: Yes.

The GPs in the think tank group (focus group 4) explicitly discussed how attending to their personal bodily responses could aid in detecting patients’ stories. The entire group resonated with this idea. One think tank GP explained:

I work a lot with using the emotions that the patient can evoke in me. So, when I notice a tenderness in myself and a tear forming in the corner of my eye, I take it as a clue that I need to pause and do something.

This quote illustrates how being mindful of one’s own emotions can guide when to give special attention to a patient’s words, thereby uncovering impactful stories. Others shared similar experiences of recognizing and intentionally using their perceptions and feelings as cues. Essentially, the think tank GPs described how they first interpreted their own emotional and physical responses before interpreting patients’ stories.

#### 4.1.2. Recognizing patterns

All GPs identified recurring patterns that they associated with impactful stories. The patterns encompassed consistent elements in patients’ life histories and symptoms and specific clusters of complaints and symptoms.

Dramatic events or trauma often emerged as *typical biographical content* in impactful stories shared with all GPs and aided them in distinguishing meaningful narratives—those notably pertinent to the patient’s health—from less substantial personal accounts. Examples include events such as losing a parent during childhood, losing one’s own child, living with severely ill parents or parents with substance abuse issues, or being exposed to severe violence or sexual abuse.

Some GPs interpreted elements of patients’ biographical accounts as representing more subtle but *demanding and often longstanding challenging life circumstances* such as stigmatization, marginalization, or rejection. One regular GP (focus group 3) summarized:

Those who have a tough story of rejection.

Other examples in this category involved a fundamental lack of feeling safe in a household with an unpredictable and often threatening atmosphere. One regular GP (focus group 3) explicitly referred to the long-lasting, detrimental impact of losing the fundamental sense of safety early in life:

Breaking a child’s trust is indeed very serious. It goes without saying. It leaves a lasting impact.

Similarly, certain *family constellations* characterized by unstable, disrupted, and/or dysfunctional relationships, including what some GPs (focus group 1) referred to as “social heritage” were also linked to impactful stories. Two participants elicited agreement in their respective group discussions as they referred to disease-ridden families who had, for two or more generations, lived with very scarce financial resources and received help from social services and sometimes also child welfare services. Some patients raised under such circumstances had also, from a very early age, carried caring responsibilities for poorly functioning parents and households.

In addition to recognizing patterns from patients’ biographies and households, regular GPs drew attention to how specific patient complaints and symptoms could represent patterns. They often hypothesized that impactful life experiences had contributed when their patients presented with symptoms like widespread chronic muscular pain, recurring headaches, fatigue, and anxiety. One regular GP (focus group 3) referred to this pattern as “a cocktail of symptoms”. Others applied terms like “symptoms that cannot be explained” or “pain from top to toe”. They typically reasoned in the manner demonstrated in the following excerpt by another regular GP (focus group 3):

If over time we investigate somewhat diffuse symptoms, like pain or other complaints where we find nothing, it makes me wonder: “Is there a story behind all this?”

#### 4.1.3. Pattern deviations

Pattern deviations entail an unusualness or divergence from an expected or typical pattern. Some regular GPs particularly the think tank GPs, provided examples of how this phenomenon could emerge during consultations. One regular GP referred to it as a feeling that something does not add up: and interpreted it as an indication that important information, like an impactful story, had not yet been revealed. One think tank GP (focus group 4) explained how encountering a logic incoherence sparked her curiosity and motivated her to look out for impactful stories:

I listen to my curiosity. When there is something in what the patient tells me that doesn’t quite make sense, it’s often important to ask about what’s going on or what has happened, because it’s often significant for the patient or for what we are going to talk about.

Several of the GPs in the think tank group pointed out how mismatches between patients’ presented complaints and their emotional responses to their illness would alert them to explore impactful stories. One think thank GP (focus group 4) elaborated on that:

The affective level doesn’t match what I would expect with what is otherwise on the agenda. The patient is panicking about a sore throat, even though there is almost nothing to find upon examination (…). I most frequently discover a significant story, in the cases where I think the patient is too emotionally affected compared to what I would expect.

Another example of pattern deviation brought up by a think tank GP was the observation that a patient had, somewhat *unexpectedly, brought along a person to take part in the consultation*:

The question is when we should consider the story important. For example, when someone brings another person to the consultation, such as a young girl bringing her mother, or a young man coming with his girlfriend. Is it because you feel uncomfortable in this situation? Is it because you can’t tell the story yourself? Is it too burdensome?

The quote above demonstrates how observing something that would appear normal in one setting, might prompt reflections and questions in the GP in other situations.

#### 4.1.4. Engaging in broad exploration

The think tank GPs specifically outlined a deliberate method for letting patients’ impactful stories unfold. It was characterized by the following features: a particular readiness, keeping quiet to let the patient talk uninterruptedly, making eye contact, and maintaining a mindful presence. One think tank GP (focus group 4) asserted that: I cannot do it if I have not chosen to be fully and completely present. I believe that is the most important tool for me.

Secondly, think tank GPs described how they deliberately assembled fragments of information and observations without jumping to conclusions on what might be troubling the patient, but rather to open up to multiple perspectives and interpretations. This kind of broad exploration proved to be an effective means of uncovering impactful stories and also contributed to forming a more comprehensive impression and understanding of such stories. One GP from the think tank (focus group 4) applied the term “tracks” to denote the various sources of information:

Of course, as a doctor, I need to figure out how to help the patient from a medical perspective. But when it comes to all the other things that happen during the consultation, like, noticing my own feelings and sensations, listening to what the patient says and has noticed, and how they interpret it, I sort that information into multiple tracks in my consciousness.

### 4.2. GPs’ involvement in impactful stories: instrumental utility or intrinsic value

The second main theme describes GPs’ aims and strategies when working with patients’ impactful stories, highlighting how their approaches seemed rooted in their perceptions of the purpose of these stories—whether as an instrumental utility or an intrinsic value. These approaches were also to a varying degree inspired by established therapeutic traditions.

#### 4.2.1. Instrumental utility

Some regular GPs consistently advocated for what we have labelled the instrumental utility of stories, meaning that the purpose of working with impactful stories was to achieve specific outcomes.

One voice within the regular GP group was represented by GPs who favoured an approach *to bring about change*, that is: to alter negative thoughts and behaviour. As one regular GP (focus group 2) explained:

When the patient has told their story, change is necessary. They must agree on “what do we do about this then?” (…) The conversation must have a purpose.

Another regular GP also inspired by facilitating change, reasoned similarly (focus group 3):

I set aside difficult patient stories that I cannot do anything about.

Both excerpts indicate that according to GPs adopting this perspective, working with the impactful stories of patients involves the specific task of addressing and resolving solvable problems.

Some of the regular GPs were also inspired by therapeutic approaches that encourage positive thinking, and focus on the patients’ strong sides, as a way of dealing with their impactful stories; “think of all the good things you have achieved in life” (GP in focus group 1).

Another GP elaborated on the rationale behind the approach in the following account (focus group 2):

Change focus and look forward to something more positive. Instead of thinking “It was terrible when Mom died because then life fell apart. Then I didn’t get supper anymore”. If one carries that thought throughout life, it becomes an unbearably sad existence.

The excerpts above illustrate a method of shifting focus away from painful experiences, aiming to alleviate the suffering and negative thoughts that impactful stories can evoke. The GPs in the think tank group (focus group 4) debated how such an approach could lead to the dismissal of impactful stories. That, in consequence, could cause more suffering, as demonstrated in the following example of a silenced story:

She came to me and cried, saying that she couldn’t endure her psychologist. It was because she wasn’t allowed to talk about her grief over the fact that her mother had recently died of cancer. The psychologist believed it was better for her not to dwell and instead focus on thinking positively and looking ahead. But she needed to talk about it. The psychologist said, “I don’t want to hear any of it”, because her own father passed away of cancer four years ago.

This example evoked both verbal and nonverbal recognition among the other think tank GPs.

#### 4.2.2. Intrinsic value of story work

All the participating GPs had experience with and were accustomed to listening to patients’ stories when they found it significant. In these cases, their approach was based on supportive and attentive listening. One regular GP (focus group 2) mentioned a meeting with a new patient who appeared unhappy. The GP understood that her sadness stemmed from losing some of her loved ones early in life. The GP elaborated on why sharing her story was important:

We concluded that she needs to describe her reality and perhaps, through that, alleviate some of the pain. Because she hasn’t told her story before.

This excerpt also demonstrates a position taken by the think tank GPs and by some of the regular GPs. It shows how *bearing witness to* impactful stories simply by listening and validating the pain has therapeutic value, even when little change is expected.

However, some of the regular GPs disagreed with that perspective. In the subsequent exchange, two of the regular GPs deliberated on the approach, referring to a colleague who allowed patients to revisit repeatedly, merely to recount the same story each time (focus group 2). One GP said:

She (the GP) says she can’t do anything about the patient’s problems, but they keep coming and keep talking about the same things.

The other GP immediately concluded:

Then days can be tough for the doctor.

Another GP ardently supported them:

One might ask whether it makes any sense for the patient to keep coming back again and again. I don’t think so.

Most of the think tank GPs and some of the regular GPs viewed the purpose of working with patients’ impactful stories as an end itself, which we have termed *the intrinsic value of story work*. These GPs’ approaches were motivated by a desire to help patients co-create a better narrative, as explained below. We have termed this *narrative co-creation*. The following excerpt illustrates this method of engaging with stories (think tank GP, focus group 4):

Now one of my patients has a father who is dying, and she is heartbroken about it. She thinks she is a bad daughter. The narrative is important here, but what kind of narrative is it? She presents the narrative that she can’t live up to life’s demands she can’t fulfil her role. But what I hear, is that she is there for her father and that she is a resourceful person. I then asked her to elaborate on that last story:” “Oh, tell me a bit more about that?” You can help her to shift the narrative to something positive, that her narrative is “I have a strength,” etc. That’s really important.

This account exemplifies how the GP starts by listening and validating the patient’s feelings and perceptions of her story. The GP then helps the patient expand and nuance the story by intentionally asking questions that encourage new perspectives. The patient and the GP explore these alternative viewpoints to deepen their understanding and interpretation. Think tank GPs also emphasized how the doctor might help patients find the words to share their stories, especially when they were confused or overwhelmed by strong emotions. This helped to create coherence in stories presented as scattered utterances and to give voice to stories that had been silenced.

### 4.3. GPs committing to impactful stories as persons—fulfilment in the face of resistance

The third main theme describes some of the particularities of general practice when GPs are accustomed to attending to patients’ impactful stories and discussing such work with their colleagues. Thus, while the two preceding themes appeared among regular and think tank GPs, this last main theme occurred almost exclusively among the think tank GPs. However, traces of it could also be found among the regular GPs, albeit in a much less explicit manner. A notable characteristic of the think tank group of GPs was their lack of ambivalence about the relevance of working with patients’ stories. When one of these GPs gave an account, they typically spoke on behalf of the entire group. This became evident as the participants frequently used the pronoun “we” and sought eye contact with other group members, who tended to nod or say “uhm” or “yes” to confirm their agreement. Discussions and minor disagreements certainly also arose among the think tank participants, but typically concerned nuances and how to best describe a complex phenomenon, that they all believed was relevant.

#### 4.3.1. The participatory mindset

As already stated, the think tank GPs took the purpose of working with patients’ impactful stories for granted. They defined this kind of work as not merely a task to be performed “now and then” in consultations when other approaches fall short. The entire group agreed that engaging with human stories broadly defines general practice: ”The story is always present in our consultations”, one of them said. Another important aspect of attending to impactful stories was the *GP participating as a person*. The following excerpt exemplifies how think tank GPs (focus group 4) articulated such active involvement:

We don’t observe patients from the outside. We meet patients and then we take part in their ongoing processes.

#### 4.3.2. Doctor’s Bliss

During the discussion within the think tank group, many GPs expressed a sense of clarity, likening it to pieces of a puzzle falling into place as they listened to and interpreted patients’ impactful stories. For them, this intellectual satisfaction had become a driving force for achieving a meaningful and fulfilling professional life. Several think tank GPs also highlighted how continuity of care for the same patient over time fostered a unique and personal doctor-patient relationship, wherein the GP gradually became intertwined with the patient’s unfolding story. One think-tank GP (focus group 4) enthusiastically recounted:

That’s a fantastic thing! The doctor is moved by the story, and then the doctor and the patient cry together.

Another described working with patients’ stories as *“doctor’s bliss”.*

Yet another GP contended that uncovering and connecting elements of patients’ stories to make them more coherent, and then helping the patient create a better narrative, gave him an immense feeling of mastery—and joy:

If I struggle during a consultation... If, for instance, my patient is a teenager who is upset, and if we finally work it out, I open my door and look at my secretary, she looks at me and immediately understands that I am happy!

#### 4.3.3. Story work under pressure

As a group, the think tank GPs experienced deep frustration over the disconnect between their belief in the central role of story work in general practice and the pressure from authorities to prioritize adherence to standardized guidelines and checklists. They believed that these organisational expectations, reflected in the lack of recognition of the value of person-centred care, undermined a central core principle of their discipline. One think tank GP (focus group 4) compared a task-focused approach to the work of a medical technician:

Do we want to be medical technicians or doctors? We can choose to become medical technicians. If we do so, we are only concerned with gathering facts and following rules. The health care system, guidelines, and the authorities encourage us to work like that.

Deliberating on their frustrations, the think tank GPs observed how the lack of established professional language and theoretical frameworks to articulate their work, hindered them, both in maturing as a collective group and in their interaction with external institutions and authorities. The following passage serves as an example. Once again, the idea of a GP participating as a person is considered crucial:

We lack the language for our complex expertise as GPs. It’s when we bring ourselves into the consultation, that we bring the expert with us – along with all that it entails. It is what we learned at university, and it is our whole being. With emotions, senses, goodwill, respect for the other (read: the patient), and the desire to help, to understand, and to make things better for that person. So - there is an incredibly complex person sitting in the doctor’s chair, meeting an equally complex human. It’s only in that relationship, that we can fully express our professional competence. And when someone tries to reduce our field to a mere fraction of what it truly is we become wildly stressed (…). But we lack the language for the complex parts of our work, and it ultimately comes down to: what is an expert in general practice?

## 5. Discussion

The present study enhances our understanding of GPs’ reflections and experiences managing patients’ impactful stories. It provides insight into various strategies GPs use to recognize and let such stories unfold. Additionally, the findings highlight the diverse purposes GPs attribute to engaging with these stories, from viewing them as instrumentally useful to recognizing their intrinsic value. These differing views influence GPs’ objectives and strategies when attending to patients’ impactful stories. Lastly, the study illuminates how a selected group of GPs find fulfilment in their commitment to patients’ impactful stories, even when they encounter external resistance.

### 5.1. Exploring the management of impactful stories

A key finding in our study is the various skills, strategies, and a specific mindset that assist GPs in managing patients’ impactful stories. Increased understanding and precision in handling such stories can help GPs attain higher levels within the triangle framework outlined in Figure 1 in the introduction. As expected, based on our selection of participants, the interviewed GPs were familiar with using renowned communication skills inspired by the patient-centred method, like attentive listening, recognizing patients’ emotions, and picking up cues and hints (Silverman et al., 2013). These skills align with level 2 in the triangle framework corresponding to Launer’s second level of narrative work in general practice (Launer, 1998).

The GPs in our study used diverse kinds of clinical reasoning as strategies to recognize impactful stories. One example is *pattern recognition*, a well-known intuitive and experiential type of diagnostic reasoning (Croskerry, 2009). Our findings extend the phenomenon from conventional medical diagnostic processes to situations where the GP senses that an impactful life story, as described in the results section, might be relevant and explain what the GP observes. This kind of inference where observations give rise to the best possible explanation or hypothesis, even if not proven, is called *abduction* (Alvesson & Sköldberg, 2018). Another example of clinical reasoning in our study is *pattern deviation*, which resonates with pattern failure: “The notion that something is different from what one would expect” (Donner-Banzhoff & Hertwig, 2014). Our study contributes to understanding how pattern deviation can arise in the context of impactful stories. These deviations may manifest as feelings evoked in the GP, observations of patients’ emotions and verbal expressions, or pre-consultation observations, such as noticing that a patient who would typically attend alone has brought an accompanying person to the consultation.

Some of the interviewed GPs outlined how they would let impactful stories emerge through broad exploration. This strategy resembles a type of clinical reasoning called *inductive foraging*, a stage preceding traditional hypothetical deductive reasoning (Donner-Banzhoff & Hertwig, 2014). The clinician deliberately conducts a broad, initial assessment to identify key points with potential relevance for further, more detailed examination. This exploratory scanning allows for considering multiple contributing factors or aetiologies underlying the patient’s problem, thereby “seeking to understand an individual’s illness in context”. Impactful stories can be among the aetiologies that inductive foraging captures.

Furthermore, looking widely at a presenting problem can prevent the physician from stopping collecting information too soon and, next, jumping to conclusions using the hypothetic deductive method, leading to “premature closure”. An example of premature closure is when a story embedded in suffering is not shared, and the patient’s symptoms are instead understood in terms of the diagnostic category of depression (Donner-Banzhoff & Hertwig, 2014). Avoiding premature closure is beneficial for promoting health, as “too often biological explanations trump biographical interpretations of patients’ problems, leading to overdiagnosis and the medicalization of human suffering” (Dowrick et al., 2016).

Another feature worth noting from our research is how the mindset of GPs can enhance their methods for handling impactful stories. GPs with the *participatory mindset* brought in their own “selves” (Dowrick et al., 2016) and appreciated the intrinsic value of impactful stories. The statement “we {GPs} always take part in the story” made by one of the think tank GPs explicitly points to how some GPs in the study viewed their role as participating persons. Another feature of the *participatory mindset* is GPs’ awareness of their bodily responses which aids in interpreting patients’ appearances, potentially linking them to impactful stories. This resembles *bodily empathy*, the embodied ability of the doctor to sense what the patient is experiencing (Rudebeck, 2000). Thus, it is an approach that extends beyond traditional clinical communication skills (Silverman et.al., 2013). McWhinney suggests something similar when discussing the need for GPs to be self-reflective and, referring to Balint, “listening to the same language in himself” (McWhinney, 2000). In our findings, the *participatory mindset* was also apparent when GPs listened to and witnessed impactful stories and established a particular way of reconstructing stories—as a joint endeavour with their patients. “Reconstructing stories” in this setting means opening to new perspectives and understandings of the story, making it “a story that works better” (Launer, 2002). Referring to the triangle presented in the introduction (Figure 1), we assign this approach to the top level.

### 5.2. Navigating dilemmas when addressing impactful stories

Our findings indicate that both patients and GPs may encounter dilemmas when addressing impactful stories. One dilemma arises in association with the differing views among GPs on the purpose of addressing and listening to impactful stories. GPs who favoured the instrumental utility of impactful stories seemed to understand their role as problem-solvers of those stories. Drawing inspiration from cognitive behavioural therapy (CBT) principles that have become popular among Norwegian and Scandinavian GPs (Haavet et al., 2023), they viewed patients’ ability to change their thoughts or behaviour as essential prerequisites for addressing impactful stories. Stories of suffering that do not easily align with the CBT approach thereby risk being overlooked or silenced. Another variant of silencing can happen as GPs too intensely emphasize patients’ strengths and positive thinking, neglecting the patient’s need to formulate a coherent story amid suffering (Brody, 1994). In instances like these, it can be argued that patients, as primary knowers of their own stories, are being subjected to *epistemic injustice*- a concept coined by philosopher Miranda Fricker, defined as “a wrong done to someone specifically in their capacity as a knower” (Fricker, 2007, p. 1).

According to Fricker, epistemic justice comes in two forms (Fricker, 2007). Relating these to our findings, the first, *testimonial injustice*, occurs when a person/patient with complex, chronic ailments is not considered trustworthy in the face of a prejudiced *listener/*clinician (Carel & Kidd, 2014). This is a phenomenon GP researchers have advised against (Aamland et al., 2017). *Hermeneutical injustice*, the second type, happens when a person lacks resources to make sense of her experiences and to *express* them in a way the clinician is ready to understand and act upon. If for instance, a GP disregards a patient’s story due to what is considered a lack of immediate informative value and coherence—what Brody (1994) might have referred to as “a broken story”—she obstructs the co-creative *development* of a coherent narrative. Hence, the patient suffers a *hermeneutical disadvantage*. Such situations arise frequently in a healthcare system dominated by a traditional biomedical discourse (Hull & Hull, 2017; Kirkengen et al., 2016).

Interestingly, our findings suggest that not only patients but also GPs can experience variants of epistemic injustice. When it comes to the GPs who were in favour of an instrumental utility of impactful stories, medical training predominantly positions doctors as problem solvers (Dowrick, 2017). This can leave GPs lacking the necessary knowledge and training to effectively engage with patients’ impactful stories (Rønneberg et al., 2022), placing them at a hermeneutical disadvantage as professional knowers (Hull & Hull, 2017). Furthermore, our study identifies another dimension of *hermeneutical disadvantage*: Some of the GPs who do feel confident in managing patients’ impactful stories often find themselves at a loss, as they lack the theoretical and linguistic resources to make their work comprehensible to other stakeholders. As it diverges from the established model of *scientific bureaucratic medicine* described by Harrison (2009), that part of their work is generally undervalued by authorities. In our study, this was explicitly discussed by the think tank GPs. It resonates with the words of Australian GP researcher Johanna Lynch who has examined the generalist philosophy of whole-person care in primary care: “Generalist care is undermined by systemic and bureaucratic devaluing of GP time and expertise, primary care policy that fragments care and encourages short transactional encounters and an excessive focus on disease or procedures” (Lynch et al., 2023, p. 428). More mundane factors also constrain GPs’ ability to engage with impactful stories. The shortage of GPs and increasing workload may compel them to deprioritize such work (Kjosavik, 2018, Rudebeck, 2019; Reeve, 2023).

### 5.3. The privilege of knowing patients’ impactful stories

Finally, one finding in our study stands out sharply, contrasting the challenges we describe above: GPs' privilege of knowing patients’ stories which fosters close relationships with their patients, and how this is a true source of meaningfulness and joy in general practice. Other studies have also highlighted the privilege of proximity (Horowitz, 2003; Molinaro et al., 2024; Scott et al., 2008). To some GPs, being close to their patients serves as “a resistance against primary care structures,” reducing the power imbalance between GPs and their patients (Molinaro et al., 2024). Along these lines, Rudebeck predicts that some GPs will always maintain close relationships with their patients, regardless of how authorities organize primary care (Rudebeck, 2019).

## 6. Strengths and boundaries

A strength of this study is that it includes the perspectives of both regular GPs and GPs with a special interest in person-centred medicine, in two neighbouring, Nordic countries. At the same time, this might also be a potential limitation, as the think tank group was Danish and the other three were Norwegian. Because the moderators of all four groups were or had been GPs themselves, we easily established contact with the participants, and we already knew the context of general practice. This may have helped participants to speak more freely. Nevertheless, our insider perspective may have caused us to miss insights that researchers from other backgrounds might offer (Dwyer & Buckle, 2009). Some of our findings might seem exclusive to the think tank group. However, this does not necessarily mean that regular GPs would not have shared similar reflections and experiences if they had been explicitly prompted during the interviews. Notably, there was little dissent among GPs in the think tank group. This could be a limitation. Additionally,we can only know what participants choose to tell. when conducting research with interview data. Another limitation of interviews as empirical data is that we only obtain descriptions and not direct observations of what participants actually do in practice.

## 7. Implications and conclusions

Drawing from evidence indicating the significant impact of life experiences on health at a fundamental level, we contend that additional professional development is needed in general practice. Our findings offer strategies and concepts that outline how work with impactful stories might be approached. Further, they can equip GPs with theoretical tools to overcome the hermeneutical disadvantage caused by uncertainty in handling significant narratives or the absence of a professional vocabulary to communicate their work to the broader community. This, in turn, fosters a deeper understanding of the importance of impactful stories and lays the foundation for integrating them as a core task in future general practice. Notably, GPs’ reflections on the “doctor’s bliss” they can experience when managing stories, highlight an uplifting aspect of their work that can counter-balance the significant strain many GPs face worldwide. Ultimately, understanding and addressing impactful stories are essential for delivering ethically sound whole-person care in general practice—care that genuinely benefits patients.

## Supplementary Material

021024 SUPPLEMENTARY MATERIAL interview guide.docx

011024 BOX 1 Stories that matter.docx

## Data Availability

The datasets generated and/or analysed during the study are not publicly available to maintain participants’ privacy but are available from the corresponding author upon reasonable request.
