# Supplementary material for: Stories that matter: a qualitative study of general practitioners’ reflections and experiences of exploring patients’ impactful life stories
Source: Int J Qual Stud Health Well-being. 2025 Jan 23;20(1):2454043. doi: 10.1080/17482631.2025.2454043 (PMC11758800; doi:10.1080/17482631.2025.2454043)
Supplement: 011024 BOX 1 Stories that matter.docx [file ZQHW_A_2454043_SM7501.docx]

***BOX 1. Medically impactful stories***

We use the term *impactful stories* as a shorthand for *stories that impact the patient’s/person’s health in a clinically significant manner, down to a deep biological level.* This refers to life events and experiences with causal relevance for the individual’s susceptibility to health problems and suffering in a wide sense, encompassing both physical and mental dimensions of health. Here, we lean on Eric Cassell’s definition of suffering as “the state of severe distress associated with events that threaten the intactness of the person”. Suffering is a subjective experience. It “can occur to any aspect of the person, whether in the realm of self, body, or family or the relation with a transpersonal, transcendent source of meaning”. Another important aspect of suffering is that it persists until “the threat of disintegration has passed or until the person can be restored in some other manner” (Cassell, 1982).

When we use the term *impactful story*, we would like to clarify that neither the doctor nor the patient can know in advance if a story will be impactful to health. Presumptions regarding a story’s medical relevance can however be based on empirical research from several disciplines, demonstrating different pathways through which life experiences affect the development of health and disease (Getz et al., 2011)

Australian GP researcher Johanna Lynch has described relevant examples of impactful stories as “Adverse childhood experiences with proven physiological impact” (Lynch, 2020, p.61) with reference to key publications in the field (Felitti, 1998; Teicher, 2015). Lynch outlines three applicable types of such experiences: *Disconnection* in the sense of being rejected and excluded. This includes both physical and emotional neglect and abuse, verbal as well as non-verbal. The second category involves physical and sexual *violence and abuse*, being a witness to violence between parents or siblings, and peer physical bullying. The last category is called the *missing parent* and points to parents who are absent, intoxicated, hospitalized, or incarcerated (Lynch, 2020, p.61).
